# *Helicobacter pylori cagA*+ Is Associated with Milder Duodenal Histological Changes in Chilean Celiac Patients

**DOI:** 10.3389/fcimb.2017.00376

**Published:** 2017-08-23

**Authors:** Yalda Lucero, Amaya Oyarzún, Miguel O'Ryan, Rodrigo Quera, Nelly Espinosa, Romina Valenzuela, Daniela Simian, Elisa Alcalde, Claudio Arce, Mauricio J. Farfán, Alejandra F. Vergara, Iván Gajardo, Jocelyn Mendez, Jorge Carrasco, Germán Errázuriz, Mónica Gonzalez, Juan C. Ossa, Eduardo Maiza, Francisco Perez-Bravo, Magdalena Castro, Magdalena Araya

**Affiliations:** ^1^Hospital Dr. Luis Calvo Mackenna Santiago, Chile; ^2^Department of Pediatrics and Pediatric Surgery, Faculty of Medicine, University of Chile Santiago, Chile; ^3^Microbiology and Micology Program, Faculty of Medicine, University of Chile Santiago, Chile; ^4^Laboratory of Immunegenetics, Institute of Nutrition and Food Technology, University of Chile Santiago, Chile; ^5^Millenium Institute of Immunology and Immunotherapy, Faculty of Medicine, University of Chile Santiago, Chile; ^6^Department of Gastroenterology, Clínica Las Condes Santiago, Chile; ^7^Hospital Militar Santiago, Chile; ^8^Hospital Dr. Roberto del Río Santiago, Chile; ^9^Clínica Las Lilas Santiago, Chile; ^10^Nutrition Program, Faculty of Medicine, University of Chile Santiago, Chile

**Keywords:** celiac disease, potential celiac disease, *Helicobacter pylori*, *cagA* gene, duodenal atrophy

## Abstract

**HIGHLIGHTS**

- What is already known about this subject?
- Celiac disease (CD) has a high clinical and histological diversity and the mechanisms underlying this phenomenon remain elusive.
- *H. pylori* is a bacterium that chronically infect gastric and duodenal mucosa activating both a Th1/Th17 and T-reg pathways.
- The role of *H. pylori* (and the effect of their virulence factors) in CD have not yet completely elucidated.
- What are the new findings?
- *cagA*+ *H. pylori* strains are associated to milder histological damage in infected CD patients.
- In active-CD patients the presence of *cagA*+ *H. pylori* is associated to an increase in T-reg markers, contrasting with a downregulation in *cagA*+ infected potential-CD individuals.
- How might it impact on clinical practice in the foreseeable future?
- The identification of microbiological factors that could modulate inflammation and clinical expression of CD may be used in the future as preventive strategies or as supplementary treatment in patients that cannot achieve complete remission, contributing to the better care of these patients.

**Background:** Mechanisms underlying the high clinical and histological diversity of celiac disease (CD) remain elusive. *Helicobacter pylori* (Hp) chronically infects gastric and duodenal mucosa and has been associated with protection against some immune-mediated conditions, but its role (specifically of *cagA*+ strains) in CD is unclear.

**Objective:** To assess the relationship between gastric Hp infection (*cagA*+ strains) and duodenal histological damage in patients with CD.

**Design:** Case-control study including patients with active-CD, potential-CD and non-celiac individuals. Clinical presentation, HLA genotype, Hp/*cagA* gene detection in gastric mucosa, duodenal histology, Foxp3 positive cells and TGF-β expression in duodenal lamina propria were analyzed.

**Results:** We recruited 116 patients, 29 active-CD, 37 potential-CD, and 50 non-CD controls. Hp detection was similar in the three groups (~30–40%), but *cagA*+ strains were more common in infected potential-CD than in active-CD (10/11 vs. 4/10; *p* = 0.020) and non-CD (10/20; *p* = 0.025). Among active-CD patients, Foxp3 positivity was significantly higher in subjects with *cagA*+ Hp+ compared to *cagA-* Hp+ (*p* < 0.01) and Hp- (*p* < 0.01). In cagA+ Hp+ individuals, Foxp3 positivity was also higher comparing active- to potential-CD (*p* < 0.01). TGF-β expression in duodenum was similar in active-CD with *cagA*+ Hp+ compared to Hp- and was significantly downregulated in *cagA*+ potential-CD subjects compared to other groups.

**Conclusion:** Hp infection rates were similar among individuals with/without CD, but infection with *cagA*+ strains was associated with milder histological damage in celiac patients infected by Hp, and in active-CD cases with higher expression of T-reg markers. Results suggest that infection by *cagA*+ Hp may be protective for CD progression, or conversely, that these strains are prone to colonize intestinal mucosa with less severe damage.

## Introduction

Celiac disease (CD) is an immune-mediated systemic disorder triggered by ingested gluten and related prolamines in genetically susceptible individuals (Husby et al., 2012; Green et al., 2015). CD has progressively increased in prevalence and is associated with significant morbidity, impacting quality of life and health care costs (Biagetti et al., 2015; Burden et al., 2015; Catassi et al., 2015). Overall prevalence is nearly 1%, ranging from 0.3 to 5% in different regions (Catassi et al., 2015; Green et al., 2015). In Chile, a National Health Survey performed in 2009 assessed a representative sample of individuals older than 15 years of age using anti-transglutaminase (tTG) antibodies detection and estimated a prevalence of 0.8% (Minsal Chile, 2010). Initially considered a gluten dependent enteropathy, CD is currently recognized as a systemic autoimmune disease with clinical manifestations ranging from asymptomatic to malabsorptive chronic diarrhea associated with severe malnutrition (Husby et al., 2012; Agardh et al., 2015; Green et al., 2015; Schøsler et al., 2016). Likewise, the intensity of inflammation and damage of the intestinal mucosa in untreated patients varies widely, from the absence of any detectable lesions to total mucosal atrophy (Donaldson et al., 2008; Husby et al., 2012). Mechanisms underlying this wide clinical and histological spectrum remain elusive.

The intestinal microbiota is currently under intense evaluation as a potentially relevant pathogenic factor in CD. Differential microbiota patterns in duodenal biopsies and stool samples have been described for CD patients as compared to healthy controls (Collado et al., 2008, 2009; Di Cagno et al., 2011; Cenit et al., 2015). *Helicobacter pylori* (*H. pylori*), a Gram-negative, spiral bacterium capable of infecting human gastric mucosa, has also been isolated from the duodenum (Csendes et al., 1995; Kim et al., 2004; Nagasawa et al., 2009). There is evidence that *H. pylori* is associated with an increased regulatory T cell (T-reg) response in blood and gastric mucosa, mainly in children, and specifically with higher number of Foxp3+ T cells and higher expression of Foxp3 and TGF-β mRNA/protein among other markers (Lundgren et al., 2005; Gil et al., 2014; Yang et al., 2014). In particular, CagA, a pleomorphic virulence factor of *H. pylori*, involved in oncogenesis and epithelial barrier disfunction, has also been involved in T-reg pathway activation (Kido et al., 2011) (reviewed in Stein et al., 2013). Meta-analysis presented in recent systematic reviews suggest a protective role of *H. pylori* against atopy, an immune mediated disorder (Lionetti, 2014; Taye et al., 2015), and some studies have proposed a modulator role in CD. Lebwohl et al. reported a strong inverse association between *H. pylori* presence in gastric mucosa and CD in a large cross-sectional study (Lebwohl et al., 2013). However, these findings have not been reproduced elsewhere (Jozefczuk et al., 2015; Simondi et al., 2015). Although, Simondi et al. did not find a difference in the prevalence of *H. pylori* between celiac and non-celiac individuals, they described an association of the presence of this bacterium in the gastric mucosa of celiac patients with milder duodenal lesions, suggesting *H. pylori* plays an immune regulator role (Simondi et al., 2015). Konturek et al. described lower seroprevalence of anti-*cagA* antibodies in celiac patients compared to healthy controls, suggesting a possible protective role of *H. pylori* harboring specific virulence factors (Konturek et al., 2000).

We aimed to determine, using a case-control design, whether *H. pylori* gastric infection, specifically with *cagA*+ strains, is associated with reduced rates of duodenal atrophy in celiac patients. Our secondary aimwas to compare duodenal expression of representative T-reg markers among CD individuals with and without infection by *cagA*+ *H. pylori* and different histological severity. Our hypothesis was that infection with *cagA*+ *H. pylori* would be more common among individuals with milder forms of CD and associated with an increase in expression of T-reg markers.

## Methods

### Study design

In a multicenter, prospective study performed in six hospitals in Santiago, Chile, subjects 1–50 years of age evaluated by their treating gastroenterologists for symptoms suggestive of CD according to clinical judgment, were invited to participate in this study. Individuals older than 50 years of age were excluded in order to reduce the possibility of recruiting patients with gastric cancer. After informed consent (and assent when appropriate) subjects were tested for celiac serology (anti-tTG) and HLA typing. When anti-tTG was positive, patient was submitted to upper gastrointestinal endoscopy (UGE) in order to obtain gastric and duodenal biopsies. Age-matched non-CD patients undergoing UGE for chronic abdominal pain or chronic diarrhea were recruited in parallel and also subject to CD serology, gastric, and duodenal biopsies.

All patients answered a survey including questions related to family history, demographics, and gastrointestinal symptoms lasting at least 1 month during the previous year. Exclusion criteria were active upper gastrointestinal bleeding, therapeutic endoscopy, use of antimicrobials or proton pump inhibitors during the previous 4 weeks, confirmed allergic enteropathy, coagulopathy, cancer, immune-suppression or inflammatory bowel disease.

### Ethics

This study was carried out in accordance with the recommendations of the Declaration of Helsinki. The protocol was approved by the IRBs of Faculty of Medicine, Universidad de Chile and the six participating centers (Hospital Dr. Luis Calvo Mackenna, Hospital Dr. Roberto del Río, Hospital Militar, Clínica Las Condes, Clínica Las Lilas, Institute of Nutrition, and Food Technology). Written informed consent was obtained from all subjects >18 years of age. In children, written informed consent was obtained on behalf of them from their Parents/Legal Guardians and in those >8 years of age, written informed assent was also obtained.

### Procedures

#### Diagnosis of CD

##### CD serology

Total IgA and IgA anti-tTG were determined in serum by a commercial ELISA kit (Alpco®, USA and AESKU®, Germany, respectively) following the manufacturer's instructions. When total serum IgA was below the cut off value for the patient's age and IgA anti-tTG was negative (< 12 UI/ml) or in a weak positive range (12–18 UI/ml), IgA/IgG anti-tTG was measured using the Celicheck kit (AESKU®, Germany). CD serology was considered positive when IgA anti-tTG was >18 UI/ml in patients with normal levels of total IgA and/or IgA/IgG anti-tTG was >24 UI/ml.

##### duodenal histopathology

At least four duodenal biopsy specimens, including a bulb sample were paraffin embedded; 4 micron sections were stained with hematoxylin-eosin and graded by an expert pathologist, blind to anti-tTG and *H. pylori* status, following the Marsh-Oberhuber classification (Marsh, 1989).

##### HLA-DQ genetic studies

HLA haplotype determination was performed to fully characterize the CD population and specifically to strength the diagnostic evidence of potential CD cases. HLA haplotypes were determined in blood using the commercial PCR kit DQ-CD Typing plus (BioDiagene®, Italy) as previously described (Araya et al., 2015). Results were expressed according to three categories: DQ2/DQ2 or DQ2/DQ8; DQ8-DQ8 or DQ8-DQ7; or non-identifiable with the kit used.

#### Histology, *H. pylori*, and *cagA* detection in gastric mucosa

Rapid urease testing was performed on antral and corporal biopsies obtained during endoscopy, using the Pronto-Dry® test (MIC France, France). Results were considered positive when color change from yellow to pink was evident, according to the manufacturer's instructions. Formalin-fixed paraffin-embedded antral and corporal biopsies were stained with hematoxylin-eosin and Warthin Starry for routine histological evaluation and *H. pylori* detection, respectively. Gastric histological damage was categorized according to the Sydney system (Stolte and Meining, 2001). Total DNA from gastric biopsies was extracted with the QIAamp RNA/DNA minikit (Qiagen Sciences, Germany) and quantified by spectrophtometry. To verify *H. pylori* DNA presence in gastric samples, amplification was performed by real time PCR using the commercial kit, Sacace™ *H. pylori* Real-TM (Sacace Biotechnologies Srl, Italy). DNA template quality was confirmed in negative samples by actin gene amplification (Genesig kit®, Primer Design, United Kingdom). The *cagA* gene was assessed in *H. pylori* positive individuals by real-time PCR of gastric tissue, as previously described (Sepúlveda et al., 2012). In brief, we used the Fast EvaGreen dye qPCR master mix (Biotium, Hayward, CA) with 4 μl of total purified DNA from each sample and the following primer pair: forward: 5- ATAATGCTAAATTAGACAACTTGAGCGA-3′ and reverse: 5′-TTAGAATAATCAACAAACATCACGCCAT-3′(Sepúlveda et al., 2012). Negative (water) and positive (ATCC 26695 reference strain) controls were used in each assay for *H. pylori* and cagA detection.

#### Classification of CD and *H. pylori* infection status

Based on the presence of anti-tTG antibodies and the Marsh-Oberhuber score, patients were classified in three groups: active-CD (positive anti-tTG antibodies and duodenal atrophy with a Marsh score of 3), potential-CD (positive anti-tTG antibodies and a Marsh score of 0–1, indicating absence of duodenal villi atrophy) and non-CD patients (negative anti-tTG and the absence of duodenal atrophy). CD patients with a Marsh score of 2 (crypt hyperplasia with no villous atrophy) were excluded in order to avoid possible ambiguity in the disease severity criteria.

A subject was considered infected by *H. pylori* when the rapid urease test, Warthin Starry staining and *H. pylori* real-time PCR were all positive in gastric samples.

#### Evaluation of T-reg markers in duodenal samples

We assessed the quantity of regulatory T-cells in duodenal mucosa of patients with and without *cagA*+ *H. pylori* by measuring the number of Foxp3+ stained cells in lamina propria by immunohistochemistry. Sections of paraffin embedded duodenal biopsies from six randomly selected patients per group (or all those available for patient groups with <6 individuals) were incubated in Dako Autostainer™ with anti-human Foxp3 (1:50, Santa Cruz), following the manufacturer's instructions. The Envision™ system (Dako) was used to visualize staining of specimens counterstained with hematoxylin eosin. For each patient we calculated the mean number of stained cells in lamina propria per 10 high power fields for three different areas of the duodenal biopsies.

For duodenal TGF-β1 measurements, we extracted total protein and RNA from mucosal samples preserved in RNA later™ using the AllPrep DNA/RNA/protein Mini Kit™ (Qiagen, Germany) following manufacturer instructions. Total RNA was reverse-transcribed by using AffinityScript™ qPCR cDNA kit (Stratagene). Synthesized cDNA was used to amplify TGF-β1 and actin genes in duplicate using TaqMan® Gene Expression Assays (Applied Biosystems™, USA). TGF-β1 expression levels were normalized to actin by subtracting the change in cycle threshold value (ΔCt). Relative expression for specific patient groups was calculated as the ratio of their mean ΔCt divided by ΔCt of active-CD patients negative for *H. pylori* (ΔΔCt).

TGF-β1 protein concentrations from duodenal biopsies were determined by Luminex, according to manufacturer's instructions and normalized by μg of total protein measured by spectrophotometry.

#### Statistical analysis

Categorical variables were compared between groups using the Chi-squared or Fisher's exact test; continuous variables were compared using the Mann-Whitney U test. The Odds ratio of the association between severity of duodenal atrophy and the presence of cagA+ *H. pylori* strains was calculated. A *p* ≤ 0.05 was considered statistically significant. For sample size calculations, we considered an expected *H. pylori* prevalence of 40% based on previous reports on the young Chilean population (Porras et al., 2013; O'Ryan et al., 2015) and an overall frequency of *cagA*+ strains of 40–80% (Harris et al., 2003; O'Ryan et al., 2015). We hypothesized a 20% detection rate of cagA+ strains in active-CD, 50% in non-CD and 80% in potential-CD patients. Twenty five cases per group would provide 80% power with a type-I error of 0.05 for a two-sided analysis.

## Results

### Patients

**Figure 1:**
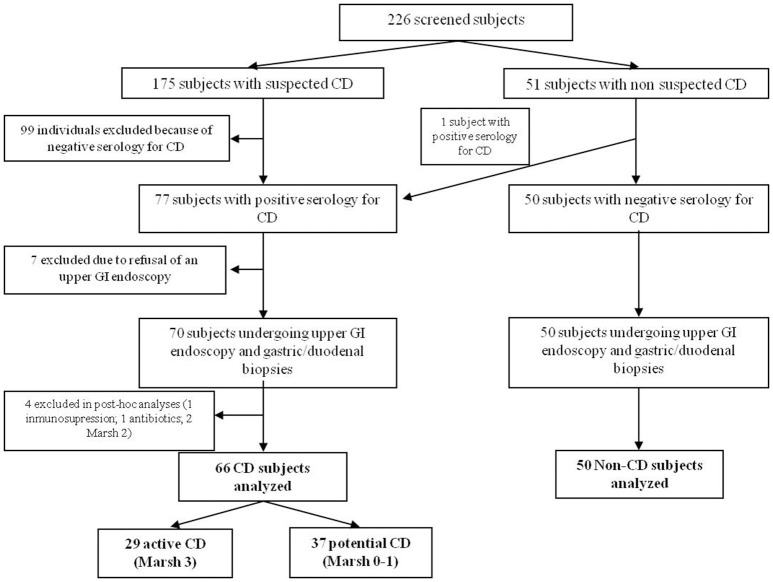
Study flow chart.

A total of 226 individuals were screened between January 2013 and July 2015, of which 116 fulfilled the inclusion criteria, provided complete data and underwent analysis; 66 were CD patients (29 with active-CD and 37 with potential-CD) and 50 were non-CD patients (Figure 1).

CD and non-CD groups were comparable by age, gender and most clinical symptoms, with the exception of abdominal pain and recent weight loss, which were significantly more frequent in non-CD patients (Table 1).

**Table 1:** Host characteristics, *H. pylori* and *cagA* detection in gastric mucosa of 66 CD patients compared to 50 non-CD individuals.

**Characteristic**		**CD patients *n* = 66**	**Non-CD patients *n* = 50**	***p*-value**
Median age, years (IQR)		12 (6–31)	14 (5–30)	0.72
Female, n (%)		40 (61)	32 (64)	0.35
Clinical presentation, n (%)	**Abdominal pain, n (%)**	**26 (39)**	**31 (62)**	**0.012**
	Bloating, n (%)	26 (39)	26 (52)	0.09
	Diarrhea, n (%)	22 (33)	20 (40)	0.23
	Constipation, n (%)	19 (29)	11 (22)	0.21
	**Recent weight loss, n (%)**	**8 (12)**	**14 (28)**	**0.017**
	Underweight, n (%)	8 (12)	11 (22)	0.08
	Vomits, n (%)	6 (9)	6 (12)	0.31
	Steatorrhea, n (%)	5 (8)	5 (10)	0.44
*H. pylori* positive, n (%)		21 (32)	20 (40)	0.18
*cagA* infected individuals		14 (21)	10 (20)	0.44
*cagA* positive/*H. pylori* positive, n (%)		14/21 (67)	10/20 (50)	0.15

*Bold values indicate significant difference (p < 0.05)*.

Age distribution, the presence of other autoimmune conditions and a family history of CD were similar between potential- and active-CD patients (Table 2). A trend toward higher frequency of DQ2/DQ2 and DQ2/DQ8 haplotypes, the presence of abdominal distension and steatorrhea was observed in the active-CD group compared to potential-CD individuals. Female gender was more frequent, anti-tTG titers were higher and the rate of constipation was significantly lower in active-CD compared with potential-CD patients (Table 2).

**Table 2:** Demographic, clinical, serological, and HLA profile of potential- and active-CD patients.

		**Potential-CD *n* = 37**	**Active-CD *n* = 29**	***p*-value**
Median age, years (IQR)		12 (5–21)	13 (7–33)	0.61
Female, n (%)		18 (49)	22 (76)	**0.01**
Other autoimmune condition, n (%)^†^		3 (8)	4 (14)	0.36
Family history of CD, n (%)		11 (30)	7 (24)	0.31
Median anti-transglutaminase levels, UI/ml (IQ range)		26.1 (18.1–51.4)	191.4 (65.4–332.7)	<**0.0001**
HLA haplotypes, n (%)	DQ2/DQ2 or DQ2/DQ8	31 (84)	28 (97)	0.09
	DQ8/DQ8 or DQ2/DQ7	4 (11)	1 (4)	
	Non-identified	2 (5)	0	
Clinical presentation, n (%)	Diarrhea	11 (30)	13 (45)	0.15
	Steatorrhea	1 (3)	5 (17)	0.05
	Constipation	14 (38)	5 (17)	**0.03**
	Abdominal distension	13 (35)	15 (52)	0.09
	Abdominal pain	14 (38)	12 (41)	0.38
	Vomiting	3 (8)	4 (14)	0.36
	Underweight	5 (14)	4 (14)	0.62

*Bold values indicate significant difference (p < 0.05)*.

### *H. pylori* and *cagA* gene detection in gastric mucosa

The frequency of *H. pylori* detection in gastric mucosa did not differ between non-CD (20/50, 40%), potential-CD (11/37, 30%) and active-CD patients (10/29, 34%) (Tables 1, 3). *cagA* gene detection in gastric mucosa of individuals infected with *H. pylori* was similar when comparing overall CD and non-CD patients (Table 1). However, in CD patients infected by *H. pylori, cagA* detection was significantly more common among individuals with potential (Marsh 0–1) compared to active-CD (Marsh 3) (*p* = 0.02; OR 15.0, 95% CI: 1.3–167.7) (Table 3). This association with *cagA*+ strains was also significant when comparing *H. pylori* infected individuals with potential-CD and non-CD group (*p* = 0.02; OR 10.0, 95%CI 1.1–93.4).

**Table 3:** *H. pylori* and *cagA* detection in patients with potential- and active-CD.

	**Potential-CD *n* = 37**	**Active-CD *n* = 29**	***p*-value**	**OR (95% CI)**
*H. pylori* positive, n (%)^*^	11 (30)	10 (34)	0.34	0.80 (0.28–2.28)
*cagA* positive, n (%)	10 (27)	4 (14)	0.15	2.31 (0.64–8.33)
*cagA* positive/ *H. pylori* positive, (%)^**^	**10/11 (91)**	**4/10 (40)**	**0.02**	**15.0 (1.34**–**167.65)**

* *A subject was considered positive for H. pylori when rapid urease test, Warthin Starry staining and real time PCR in gastric mucosa were all positive*.

** *comparison of cagA detection rate in patients infected by H. pylori*.

*Bold values indicate significant difference (p < 0.05)*.

In order to characterize the effect of *H. pylori* infection at the gastric level in our study population we compared histology results according to infection status. Overall histological gastritis was more common in non-CD subjects infected with *H. pylori* compared to non-infected patients (*p* = 0.003; OR 7.6, 95%CI 1.7–38.2); however there was only a trend toward increased gastritis in *H. pylori* positive individuals in the potential-CD (*p* = 0.069) and active-CD groups (*p* = 0.091) (Table 4). There was no association between histological gastritis and *cagA*+ strains (Table 4). We identified only three cases of atrophic gastritis (all in *H. pylori* negative individuals) and two cases of intestinal metaplasia (one in an *H. pylori* negative and one in an *H. pylori* positive subject) (Table 4).

**Table 4:** Gastric histological findings according to CD and *H. pylori* (*Hp*) status.

	**Potential CD** ***n*** = **37**			**Active CD** ***n*** = **29**			**Non-CD patients** ***n*** = **50**			***p*-value**
	***Hp* + *cagA* + *n* = 10**	***Hp* + *cagA* −*n* = 1**	***Hp* −*n* = 26**	***Hp* + *cagA* + *n* = 4**	***Hp* + *cagA* −*n* = 6**	***Hp* −*n* = 19**	***Hp* + *cagA* + *n* = 10**	***Hp* + *cagA* −*n* = 10**	***Hp* −*n* = 30**	
Normal	1	1	13	0	1	9	3	1	19	n.s.
Mild chronic gastritis	7	0	11	4	1	9	3	6	10	n.s.
Moderate chronic gastritis	2	0	0	0	3	0	4	3	0	n.s.
Severe chronic gastritis	0	0	0	0	0	0	0	0	0	n.s.
Atrophic gastritis	0	0	1	0	0	1	0	0	1	n.s.
Intestinal metaplasia	0	0	1	0	1	0	0	0	0	n.s.

### Foxp3 positive cell count and TGF-β1 expression in duodenal biopsies according to *H. pylori* and cagA status

**Figure 2:**
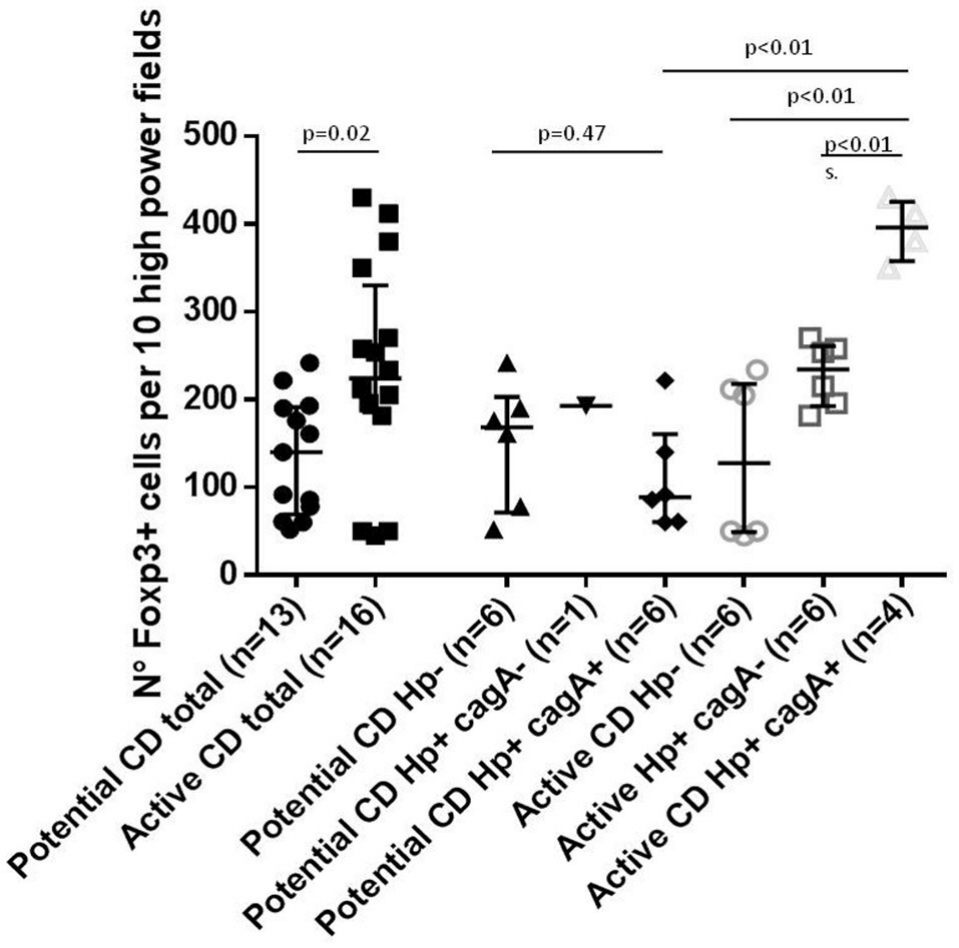
Number of Foxp3 positive cells per 10 high power field in lamina propria stained by immune histochemistry.

Considering the association between cagA+ *H. pylori* strains and milder histological damage in CD subjects, we assessed levels of selected T-reg markers in order to explore if this pathway could be involved in the halting of disease progression. Overall the median number of Foxp3 positive cells in lamina propria was significantly higher in patients with active- compared to potential-CD (Figure 2). In the group of potential-CD subjects there was no difference between *H. pylori* infected and non-infected (Figure 2). In patients with active-CD infected with *H. pylori* cagA+ the number of Foxp3 positive cells was significantly higher compared to all the other active-CD groups as well as to potential-CD subjects infected by cagA+ *H. pylori* (*p* < 0.05; Figure 2).

**Figure 3:**
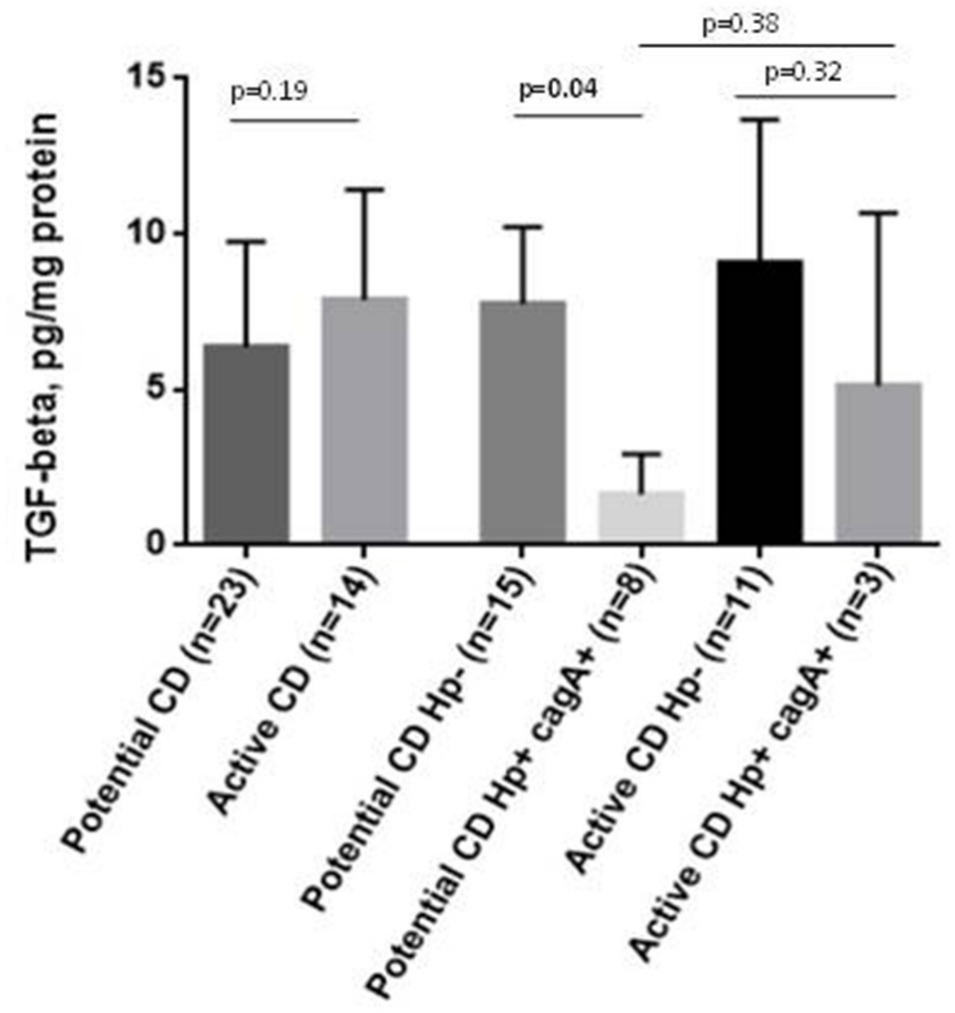
TGF-β1 in duodenal mucosa in active CD and potential CD comparing those negative for *H. pylori* and positive for *cagA*+ *H. pylori* strains in gastric mucosa. TGF-β1 levels were quantified by Luminex and expressed per mg of protein.

TGF-β1 protein concentration in duodenal biopsies showed a tendency to be higher in active-CD compared to potential CD overall (*p* = 0.19). Individuals infected by *cag*+ *H. pylori* strains did have lower TGF-β1 levels than non-infected individuals, that was statistically significant in the potential-CD group (*p* = 0.04; Figure 3). This finding was consistent with the results of duodenal TGF-β1 mRNA measurement (See Supplementary Figure 1 in Supplementary Material).

## Discussion

In this case-control study including predominantly young patients, overall *H. pylori* infection rates were similar between subjects with and without CD, and were independent of the severity of duodenal atrophy in the latter. However, in individuals infected by *H. pylori*, detection of *cagA*+ strains was significantly more common among individuals with potential-CD compared to active-CD and non-CD patients. A similar *H. pylori* prevalence between CD and non-CD subjects have been previously reported. Aydogdu et al. found a 22% of *H. pylori* prevalence in 96 Turkish CD children compared to 24% in 235 non-CD children (mean age of 8 years old; Aydogdu et al., 2008). Similar prevalence rates were reported by Luzza et al in Italian children with and without CD (Luzza et al., 1999). On the other hand, Simondi et al. determined a higher, but also similar prevalence of *H. pylori* in CD and non-CD adults (36 and 41%, respectively; mean age of 41 years old; Simondi et al., 2015). In contrast to these findings, Lebwohl et al. reported a strong inverse association between the presence of *H. pylori* in gastric mucosa and CD in a large cross-sectional study that included 136,179 patients undergoing upper endoscopy in the USA (OR = 0.48; 95%; CI: 0.40–0.58; Lebwohl et al., 2013). This study was performed in a country with a low-prevalence of *H. pylori* (prevalence 4.4 and 8.8% in CD and non-CD subjects, respectively) and included older patients compared to our clinical series (mean age 51 years old vs. 13 years old), which could in part explain the discrepancy.

Milder histological damage among CD patients infected with *H. pylori* was previously reported by Villanacci et al. (in a group of 80 CD adults *H. pylori* was detected in 7/9 individuals Marsh 1–2 and 23/71 Marsh 3; Villanacci et al., 2006), Aydogdu et al. (in a group of 96 CD children *H. pylori* was detected in 56% of Marsh 1, 38% of Marsh 2 and 5% of Marsh 3, *p* < 0.05; Aydogdu et al., 2008), and Simondi et al. (in a group of 73 CD adults *H. pylori* was detected in 50% of Marsh 1–2 and 33% of Marsh 3; *p* > 0.05; Simondi et al., 2015). Our study differs from the above mentioned because the association between *H. pylori* and CD related histological damage was the primary and not secondary outcome and we additionally explored the possible effect of a virulence factor (*cagA*). Overall *H. pylori* prevalence did not differ between potential- and active-CD patients (both nearly 30–40%); however among infected subjects, the presence of *cagA*+ strains was significantly associated with milder histological damage (Marsh 0–1). Prevalence of *cagA*+ *H. pylori* strains vary between populations between 40 and 80% (Feliciano et al., 2015; O'Ryan et al., 2015; Sayehmiri et al., 2015; Scarpulla et al., 2015) and could explain at least in part the difference observed between our study and previous findings. The presence/absence of the *cagA*+ gene among infecting *H. pylori* strains is a factor that may be modulating the severity of duodenal lesions. Konturek et al. reported that anti-CagA antibodies were detected significantly less frequently in *H. pylori*+ celiac subjects than in healthy *H. pylori*+ controls (Konturek et al., 2000), which is in line with our findings.

*H. pylori* infections, which are acquired mostly early in life in Chilean children (O'Ryan et al., 2015), may have a limited role in overall development of CD, possibly modulating disease in subsets of individuals. The fact that for potential-CD subjects infected with *H. pylori* most are infected with *cagA*+ strains, in contrast to subjects with active disease where most are infected with *cagA-* strains, suggests that infection with the former pathotype may be modulating disease to less severe forms of presentation.

Conversely, it is possible that gastric/duodenal tissue of these potential-CD individuals is more prone to be colonized with *cagA*+ strains. However, counter evidence to this hypothesis was reported in a previous Chilean cohort study in which acquisition of persistent *H. pylori* infection occurred mainly during the first 3 years of life (O'Ryan et al., 2015), an age that is 10 years younger than the median age of our series of patients. It is reasonable to extrapolate that in most of our cases, *H. pylori* infection preceded the development of CD. Whether persistently infected children change strains over time, from *cagA*+ to *cagA-* (or vice versa), remains as yet an open question requiring a longitudinal study design.

The number of positive stained Foxp3 cells in duodenal lamina propria and the expression of TGF-β1 were higher in active than in potential CD patients, consistent with findings by Tiittanen et al. (2008), Brazowski et al. (2010), and Borrelli et al. (2013). In active-CD, the T-reg pathway is fully activated downregulating the ongoing Th1/Th17 inflammatory process (Tiittanen et al., 2008). However, *in vitro* evidence suggests that effector T cells become resistant to suppression by T-regs in this setting (Hmida et al., 2012). According to our results, this increase in Foxp3 cells was even more evident in active-CD patients infected with *cagA*+ *H. pylori* strains. Although duodenal inflammatory profiles have not been previously explored in patients infected with *H. pylori*, this agent has been associated with a local gastric and systemic response characterized by Th1/Th17 inflammatory processes as well as activation of T-reg pathways contributing to infection persistence (Gil et al., 2014; Hussain et al., 2016). The T-reg pathway is especially enhanced in children compared to adults (Freire de Melo et al., 2012; Serrano et al., 2013; Gil et al., 2014) and in individuals infected with *cagA*+ *H. pylori* strains (Kido et al., 2011; Hussain et al., 2016). We hypothesize that priming with *cagA*+ *H. pylori* at a young age, may protect against chronic inflammatory processes and loss of food/self antigen tolerance through activation of the T-reg pathway.

Further studies of cytokine expression in cultured duodenal biopsies of CD patients exposed *in vitro* to *H. pylori* with and without *cagA* gene and cohort studies of high risk CD patients infected and non-infected with *cagA*+ *H. pylori* strains are needed to confirm this proposal.

Animal models fully reproducing CD are not currently available but comparing potential-CD with active-CD individuals is an interesting “*in vivo*” model to advance knowledge on mechanisms underlying this condition. The identification of factors that could modulate histological damage and inflammation in CD can potentially open new avenues for prevention and treatment aiming to improve the quality of life of these patients.

In conclusion, *H. pylori* infection rates were similar among individuals with and without CD, but infection with *cagA*+ strains was significantly associated with milder histological damage in celiac patients infected by *H. pylori* and in active-CD cases with higher expression of T-reg markers. These results suggest that infection by *cagA*+ *H. pylori* may be protective for CD progression, or conversely, that these strains are prone to colonize when intestinal damage is less severe. Future research should focus on clarifying mechanisms involved in this association.

## Author contributions

Substantial contributions to the conception or design of the work: YL, AO, MO, RQ, NE, RV, and MA. Contribution to the acquisition, analysis, or interpretation of data for the work: YL, AO, MO, RQ, NE, RV, DS, EA, CA, MF, AV, IG, JM, JC, GE, MG, JO, EM, FP, MC, and MA. Drafting the work or revising it critically for important intellectual content: YL, AO, MO, RQ, NE, RV, DS, EA, CA, MF, AV, IG, JM, JC, GE, MG, JO, EM, FP, MC, and MA. Final approval of the version to be published: YL, AO, MO, RQ, NE, RV, DS, EA, CA, MF, AV, IG, JM, JC, GE, MG, JO, EM, FP, MC, and MA. Agreement to be accountable for all aspects of the work in ensuring that questions related to the accuracy or integrity of any part of the work are appropriately investigated and resolved: YL, AO, MO, RQ, NE, RV, DS, EA, CA, MF, AV, IG, JM, JC, GE, MG, JO, EM, FP, MC, and MA.

### Conflict of interest statement

The authors declare that the research was conducted in the absence of any commercial or financial relationships that could be construed as a potential conflict of interest.

## Supplementary material

Supplementary Figure 1 TGF-β1 mRNA relative expression in active CD and potential CD comparing those negative for H. pylori and positive for cagA+ H. pylori strains in gastric mucosa. ΔCt was calculated by using actin expression as reference and normalized assuming active-CD negative for H. pylori as 1 (samples processed in duplicate).
