# Corrigendum: *Helicobacter pylori cagA*+ Is Associated with Milder Duodenal Histological Changes in Chilean Celiac Patients

**DOI:** 10.3389/fcimb.2017.00427

**Published:** 2017-09-29

**Authors:** Yalda Lucero, Amaya Oyarzún, Miguel O'Ryan, Rodrigo Quera, Nelly Espinosa, Romina Valenzuela, Daniela Simian, Elisa Alcalde, Claudio Arce, Mauricio J. Farfán, Alejandra F. Vergara, Iván Gajardo, Jocelyn Mendez, Jorge Carrasco, Germán Errázuriz, Mónica González, Juan C. Ossa, Eduardo Maiza, Francisco Perez-Bravo, Magdalena Castro, Magdalena Araya

**Affiliations:** ^1^Department of Pediatrics and Pediatric Surgery, Faculty of Medicine, University of Chile, Santiago, Chile; ^2^Microbiology and Micology Program, ICBM, Faculty of Medicine, University of Chile, Santiago, Chile; ^3^Pediatric Gastroenterology Unit, Department of Pediatrics, Clínica Alemana-Universidad del Desarrollo, Santiago, Chile; ^4^Laboratory of Immunegenetics, Institute of Nutrition and Food Technology, University of Chile, Santiago, Chile; ^5^Millenium Institute of Immunology and Immunotherapy, Faculty of Medicine, University of Chile, Santiago, Chile; ^6^Department of Gastroenterology, Clínica Las Condes, Santiago, Chile; ^7^Hospital Militar, Santiago, Chile; ^8^Hospital Dr. Luis Calvo Mackenna, Santiago, Chile; ^9^Hospital Dr. Roberto del Río, Santiago, Chile; ^10^Clínica Las Lilas, Santiago, Chile; ^11^Nutrition Program, Faculty of Medicine, University of Chile, Santiago, Chile

**Keywords:** celiac disease, potential celiac disease, *Helicobacter pylori*, *cagA* gene, duodenal atrophy

In the published article, there was an error in affiliation (2). Instead of “Microbiology and Micology Program, Faculty of Medicine, University of Chile, Santiago, Chile,” it should be “Microbiology and Micology Program, ICBM, Faculty of Medicine, University of Chile, Santiago, Chile.” The authors apologize for this error and state that this does not change the scientific conclusions of the article in any way.

Additionally, there was an error regarding the affiliations for Yalda Lucero. Affiliation 8 has been removed and the following affiliation has been added instead: Pediatric Gastroenterology Unit, Department of Pediatrics, Clínica Alemana-Universidad del Desarrollo, Santiago, Chile.

## Conflict of interest statement

The authors declare that the research was conducted in the absence of any commercial or financial relationships that could be construed as a potential conflict of interest.

