# Supplementary figures and images for: Helicobacter pylori cagA+ Is Associated with Milder Duodenal Histological Changes in Chilean Celiac Patients
Source: Front Cell Infect Microbiol. 2017 Aug 23;7:376. doi: 10.3389/fcimb.2017.00376 (PMC5572207; doi:10.3389/fcimb.2017.00376)

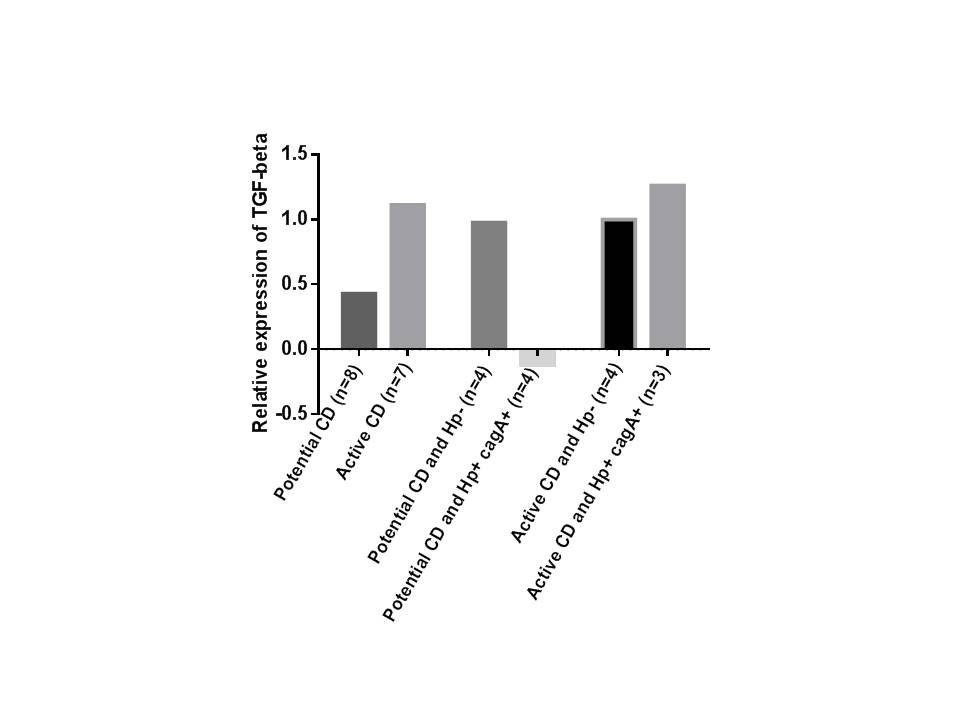

Supplement: Supplementary Figure 1 — TGF-β1 mRNA relative expression in active CD and potential CD comparing those negative for H. pylori and positive for cagA+ H. pylori strains in gastric mucosa. ΔCt was calculated by using actin expression as reference and normalized assuming active-CD negative for H. pylori as 1 (samples processed in duplicate). [file Image1.JPEG]
